# The Role of Indoleamine 2,3 Dioxygenase in Beneficial Effects of Stem Cells in Hind Limb Ischemia Reperfusion Injury

**DOI:** 10.1371/journal.pone.0095720

**Published:** 2014-04-21

**Authors:** Mohamad Masoumy, Jack Yu, Jun Yao Liu, Nathan Yanasak, Christopher Middleton, Folami Lamoke, Mahmood S. Mozaffari, Babak Baban

**Affiliations:** 1 Department of Surgery, Georgia Regents University, Augusta, Georgia, United States of America; 2 Department of Oral Biology, Georgia Regents University, Augusta, Georgia, United States of America; 3 Department of Radiology and Imaging, Georgia Regents University, Augusta, Georgia, United States of America; 4 Cancer Center, Georgia Regents University, Augusta, Georgia, United States of America; 5 Department of Ophthalmology, Georgia Regents University, Augusta, Georgia, United States of America; University Hospital of Heidelberg, Germany

## Abstract

Ischemia-Reperfusion (IR) injury of limb remains a significant clinical problem causing secondary complications and restricting clinical recovery, despite rapid restoration of blood flow and successful surgery. In an attempt to further improve post ischemic tissue repair, we investigated the effect of a local administration of bone marrow derived stem cells (BMDSCs) in the presence or absence of immune-regulatory enzyme, IDO, in a murine model. A whole limb warm ischemia-reperfusion model was developed using IDO sufficient (WT) and deficient (KO) mice with C57/BL6 background. Twenty-four hours after injury, 5×10^5^ cells (5×10^5^ cells/200 µL of PBS solution) BMDSCs (Sca1 + cells) were injected intramuscularly while the control group received just the vehicle buffer (PBS). Forty-eight to seventy-two hours after limb BMDSC injection, recovery status including the ratio of intrinsic paw function between affected and normal paws, general mobility, and inflammatory responses were measured using video micrometery, flow cytometry, and immunohistochemistry techniques. Additionally, MRI/MRA studies were performed to further study the inflammatory response between groups and to confirm reconstitution of blood flow after ischemia. For the first time, our data, showed that IDO may potentially represent a partial role in triggering the beneficial effects of BMDSCs in faster recovery and protection against structural changes and cellular damage in a hind limb IR injury setting (*P* = 0.00058).

## Introduction

Ischemia-Reperfusion injury is seen in many clinical scenarios from stroke, myocardial infarction, traumatic limb injury, embolic events and organ transplantation. According to statistics from the CDC, nearly 2 million people a year in the US are affected by the above conditions [1]. Therefore, there is no shortage of opportunity to promote the health and lives of patients under each circumstance by improving our understanding of the mechanisms by which IR injury occurs, so that modulation and attenuation of injury can be achieved for therapeutic purposes. IR injury begins with the initial ischemic event leading to decreased oxygen and nutrient delivery. The resulting metabolic derangements in affected tissues lead to a buildup of acidic metabolites, dysfunction of organelles and build of free radicals, which include reactive oxygen and nitrogen species. Additionally, leukocytes become activated releasing cytokines and complement mediators, and inflammatory cascades are activated as a result of cell necrosis.

Moreover, these structural changes markedly increase mitochondrial dysfunction secondary to the ischemic injury, leading to increased membrane permeability, influxes of calcium, uncoupling of the electron transport chain and release of ROS [2]. Collectively, the response to IR injury leads to innate and adaptive immune activation, microvascular dysfunction, severe inflammation and ultimately, cell death.

Stem cells possess the capacity to self-renew and develop into functionally specialized cells. Implanted stem cells can be integrated into various host organs, survive, and reverse different ischemic deficits [3]. Adult stem cells and bone marrow stromal cells have now shown promise in experimental models of ischemic diseases including stroke, cardiac infarction, ischemic retinopathy, and limb ischemic injury. The plasticity of stem cells and the potential to manipulate their response depending on the milieu to which they are exposed has led to stem cell therapy (SCT) being an attractive field of investigation for its many potential clinical applications including ischemia-reperfusion (IR) injury. Numerous studies have shown stem cells to not only promote angiogenesis, but also to exert anti-inflammatory effects via paracrine and autocrine signaling [4]–[8]. The crosstalk that occurs between the stem cells and the microenvironment is the key to their observed response. The cytokines, chemokines and growth factors released and the receptors expressed by stem cells during this time, alters the milieu ultimately attenuating the inflammatory response and damage from IR injury, while helping promote and enhance tissue repair and recovery [9]–[12]. Our previous studies have shown that the immunomodulator leflunomide, which pharmacologically recruits bone marrow derived stem cells (BMDSC), in addition to inhibiting the proliferation of lymphocytes, helps protect renal tissue subjected to IR injury by up-regulating interleukin (IL)-10, down regulating IL-17 and IL-23, while persevering the mitochondrial membrane potential (ψm) and reducing overall cell death [13], [14].

Indoleamine 2,3-dioxygenase (IDO) has emerged as a pivotal modulator/regulator of the immune response [13], [28], [34]. IDO is a heme-containing cytosolic enzyme that is the rate limiting catalyst to the metabolism of the essential amino acid tryptophan within the kynurenine pathway [15], [16]. These genes contain interferon (IFN) response elements and therefore, IFNs are powerful inducers of IDO expression [23]. Over the last two decades, IDO has been investigated in the fields of immunology and microbiology to help develop a better understanding of its role within eukaryotic cells beyond its role in tryptophan metabolism. More recently, studies have shown IDO expression to be important in the ability for tumor cells to be immune tolerant [17]–[19]. Additionally, IDO helps in activation of t-regulatory cells, resulting in containment of hyperinflammatory responses and inhibition of t-helper 17 cells recruitment [14]. Importantly, stem cells express the complete kynurenine pathway indicative of its functional role(s) in stem cell biology. Up regulation of IDO expression using interferon-γ within mesenchymal stem cells (MSCs) have shown to reduce inflammatory conditions [8], [20]–[22]. However, it remains to be established whether the status of stem cells with respect to IDO is a determinant of the local inflammatory response and prevention of tissue injury in the setting of IR injury. Therefore, developing a more profound understanding of the specific mechanisms by which SCT and IDO deliver their effects and how they are related will contribute to exploring the translational potential for more targeted and effective therapies in IR injury and other inflammatory conditions.

IDO is an important immune-modulator with the ability to help confer tolerance and suppression depending on the environmental milieu, and the therapeutic effects of stem cells in ischemic models. This article is to investigate whether the beneficial function of stem cells in the treatment of an ischemia reperfusion model is an IDO-dependent mechanism. To the best of our knowledge, this is the first study focused on the interaction of IDO and stem cells in a hind limb ischemia reperfusion model, which can be potentially translated into an effective therapeutic protocol in the treatment of hind limb ischemia reperfusion.

## Materials and Methods

### Animals

All animal procedures were performed in accordance with the approval of the Institutional Animal Care and Use Committee of Georgia Regents University. Male IDO sufficient wild-type (WT) and IDO deficient C57BL/6 mice (n = 5/group) were obtained (Jackson Laboratories, Bar Harbor, ME, USA).

### Bone Marrow Derived Stem Cells Isolation

Bone marrow derived stem cell (BMDSCs) was isolated from the femur and tibia of IDO WTC57BL/6 mice 10–14 weeks of age. Briefly, marrow was flushed out of the bones by inserting a 27 g connected to a 3 ml syringe (filled with medium, RPMI-1640 plus FBS 10%) into the cavity and washing out the central core of cells into a Petri dish. To homogenize, any clumps was broken up by pipetting or with the plunger of the syringe followed by harvesting the cell suspension into a 15 ml tube through 70 mm nylon mesh to remove any smaller clumps of cells. Cell suspension then was span down (1500 rpm for 5 minutes) and re-suspended in medium. Using the magnetic-activated cell sorting (MACS) technique following manufacturer’s instructions (Miltenyi Biotech, Auburn, CA), BMDSCs were separated out from total bone marrow cells by targeting Sca1 (marker for murine stem cells).

### Induction of Ischemia and Treatment

To induce the whole limb warm ischemia-reperfusion model, both the arterial inflow and venous outflow were occluded via external constriction for 90 minutes using an elastic lasso device. Twenty-four hours after inducing limb IR injury, 5×10^5^ BMDSCs were injected intramuscularly. Forty-eight to seventy-two hours after limb BMDSC injection, recovery status including ratio of toe spread between affected and normal paws, general mobility, and inflammatory responses were measured using video micrometer, and flow cytometry techniques. Additionally, MRI/MRA studies were performed to further study the inflammatory response between groups and to confirm reconstitution of blood flow after ischemia.

### Analytical Flow Cytometry

Soft tissues from the site of injury were sieved through a cell strainer (BD Biosciences, San Diego, CA), followed by centrifugation (1,500 rpm, 10 min) to prepare single-cell suspensions. Phenotypic and intracellular analyses of cells were performed as described previously [28], [38]. Briefly, samples were treated with reagents, and stained with fluorochrome-conjugated antibodies of interest based on manufacturers’ instructions. Antibodies against Sca1 (marker of stem cells), Annexin V, and 7-aminoactinomycin D (7AAD; for apoptosis/necrosis), and FoxP3 (for Tregs) were obtained from BD Biosciences (San Diego, CA). Further, IL-23, IL-17, and IL-10 antibodies were purchased from eBioScience (San Diego, CA). Next, cell samples were run through a four-color flow cytometer (FACS Calibur, BD Biosciences, San Diego, CA), and data were collected using CellQuest software. Samples were double-stained with control IgG, and cell markers were used to assess any spillover signal of fluorochromes; proper compensation was set to ensure the median fluorescence intensities of negative and positive cells were identical and were both gated populations. Gating was used to exclude dead cells and debris using forward and side scatterplots. As a gating strategy for each sample, isotype-matched controls were analyzed to set the appropriate gates. For each marker, samples were analyzed in duplicate measurements. To minimize false-positive events, the number of double positive events detected with the isotype controls were subtracted from the number of double-positive cells stained with corresponding antibodies (non-isotype control), respectively. Cells expressing a specific marker were reported as a percentage of the number of gated events.

### Immunohistochemical Analysis

Formalin-fixed paraffin-embedded soft tissues were cut in 4 µm sections and processed for immunohistochemical assessment of IDO, IL-17, IL-23, IL-10 and FOXP3 according to previously described protocols [28], [38]. All antibodies for immunohistochemistry analysis were purchased from Santa Cruz Biotechnology, USA.

### Video Micrometer/Toe Spread

A 60 sec video recording of the animal was created in a clear plastic cage with all beddings removed to allow for an unimpeded view of the extremities. At 5 random time points every 5–10 seconds, the video frame was frozen and the picture magnified and examined for quality adequacy. We used a modified version of walking track analysis to measure the functional outcome of stem cell (SC) injection in ischemia-reperfusion (IR) injury, as described by Lee *et al*. [40]. Briefly, the toe spread was measured in the IR limb (Ti) and control contralateral limb (Tc). The ratio of the toe spread of the paw of the IR limb (Ti) to the control limb (Tc) was then calculated by Ti/Tc. As shown, 100% indicated equal width and, thus normal intrinsic function. If both the right and left hind paws could be seen, then it was deemed acceptable and the width of the hind paws were measured using a micrometer and the ratio of the smaller one to the larger one was calculated. The individual performing this part of the outcome measure was blinded in regards to the identity and treatment history of the animal in the video being analyzed. Standard descriptive statistics, including mean and standard deviations, were then calculated and the inferential statistics were done using two-tailed student t-test. Alpha was set at 0.05 and with power at 80%.

### MRI/MRA Imaging Acquisition

MRI images were acquired for physiological characterization of mouse thigh and calf muscle during and after the application of a tourniquet for 90 minutes. Additionally, MRA studies were performed to confirm the occlusion and reconstitution of blood flow to validate our model. Adult male wild-type and IDO knockout C57/BL6 background mice were imaged using MRI following the guidelines of the Animal Care and Use Committee and the Animal Health and Care Section of GRU.

Initially, each mouse underwent 3 imaging sessions. They were imaged while the tourniquet was on, immediately after the tourniquet was released and 48–72 hours after injury. The purpose of performing the MRA imaging sequences was to confirm that tourniquet application occluded blood flow and that the blood flow reconstituted once the tourniquet was released. Once these initial parameters were confirmed, subsequent experimental groups were imaged 48–72 hours after their treatment was administered. There were a total of 4 experimental groups imaged: WT mice treated with stem cells (n = 4) or PBS (n = 3), and the IDO KO mice treated with stem cells (n = 4) and PBS (n = 4). The animals were anesthetized with a mixture of medical air, oxygen (1∶1), and 2.5% isoflurane. After positioning the animal on a cradle to maintain a constant body temperature (37.8°C), the animal was maintained on ∼1.25% isoflurane throughout the MRI session. For the purposes of constraining motion, the abdomen of each mouse was secured to the cradle using medical tape.

Images were acquired on a 7.0T 20 cm bore BioSpec MRI spectrometer (Bruker Instruments, Billerica, MA, USA). A standard transmit/receive volume coil (35 mm i.d.) was used for imaging. ECG and respiratory signals were monitored by a physiological monitoring system (SA Instruments, INC., Stony Brook, NY). For investigation of tissue edema, a 2D RARE sequence was used to acquire T2-weighted axial images (TE_eff_/TR = 12/2890msec; RARE Factor = 8; # of slices = 24; slice thickness = 1 mm; slice gap = 0.5 mm; # of averages = 5; Matrix = 256×256; FOV = 32.0×32.0 mm^2^). The imaging volume was set to begin just superior to the hip joint at the pelvis. For all mice, the stack of imaging slices subtended a volume reaching to the bottom of the calf. For visualization of reperfusion of major leg vessels post-tourniquet, images were collected in a subset of animals during application of and immediately subsequent to removal of a tourniquet using a 2D time-of-flight MR Angiography (2D TOF MRA) sequence (TE/TR = 4/19.5 msec; # of slices = 90; slice thickness = 0.3 mm; slice gap = 0.1 mm; # of averages = 4; Matrix = 352×320; FOV = 32.0×32.0 mm^2^). In the case of imaging during application of the tourniquet and shortly thereafter, the mouse cradle was moved out of the scanner. The tourniquet was removed with the animal attached to the cradle so that it could be relocated to a similar position for scanning post-tourniquet.

### MRI/MRA Imaging Processing

The mice from each of the 4 groups underwent T2 weighted imaging of the injured and non-injured limbs. Using Image J to process the images taken [23], measurements of the soft tissue intensity were taken from the anterior lateral, anterior medial and posterior thigh in each leg in 3 consecutive image slices distal to the level of the injury. Given that T2 weighted imaging enhances with increases in soft tissue inflammation, such measurements were used to help correlate clinical findings of response to injury and therapy. The average intensity measured from each leg was then used to create a ratio comparing the intensity of the injured leg to the contra-lateral leg. Once each ratio was obtained, the measured ratios were compared to one another to evaluate the response to the different treatments and then plotted onto representative graphs.

MRA images were processed for display and qualitative inspection of vessel clamping during application of the tourniquet, using Image J [23]. Images were loaded into the software, and vessels were manually segmented into groups including left leg, right leg, and tail. Each of these groups was stacked into separate maximum intensity projection images (MIPs). After each group was assigned a color (red, green, or blue), the MIPs were summed together into a composite image for easy inspection of vessels.

### Statistical Analysis

All data are presented as mean ± SEM. Laser Doppler measurements were analyzed using SPSS 17.0 repeated measures ANOVA and LSD post-hoc test. In all cases, p-value ≤0.05 was considered to be significant.

## Results

### Stem Cells Insert their Beneficial Effects in an IDO Dependent Fashion

**Figure 1:**
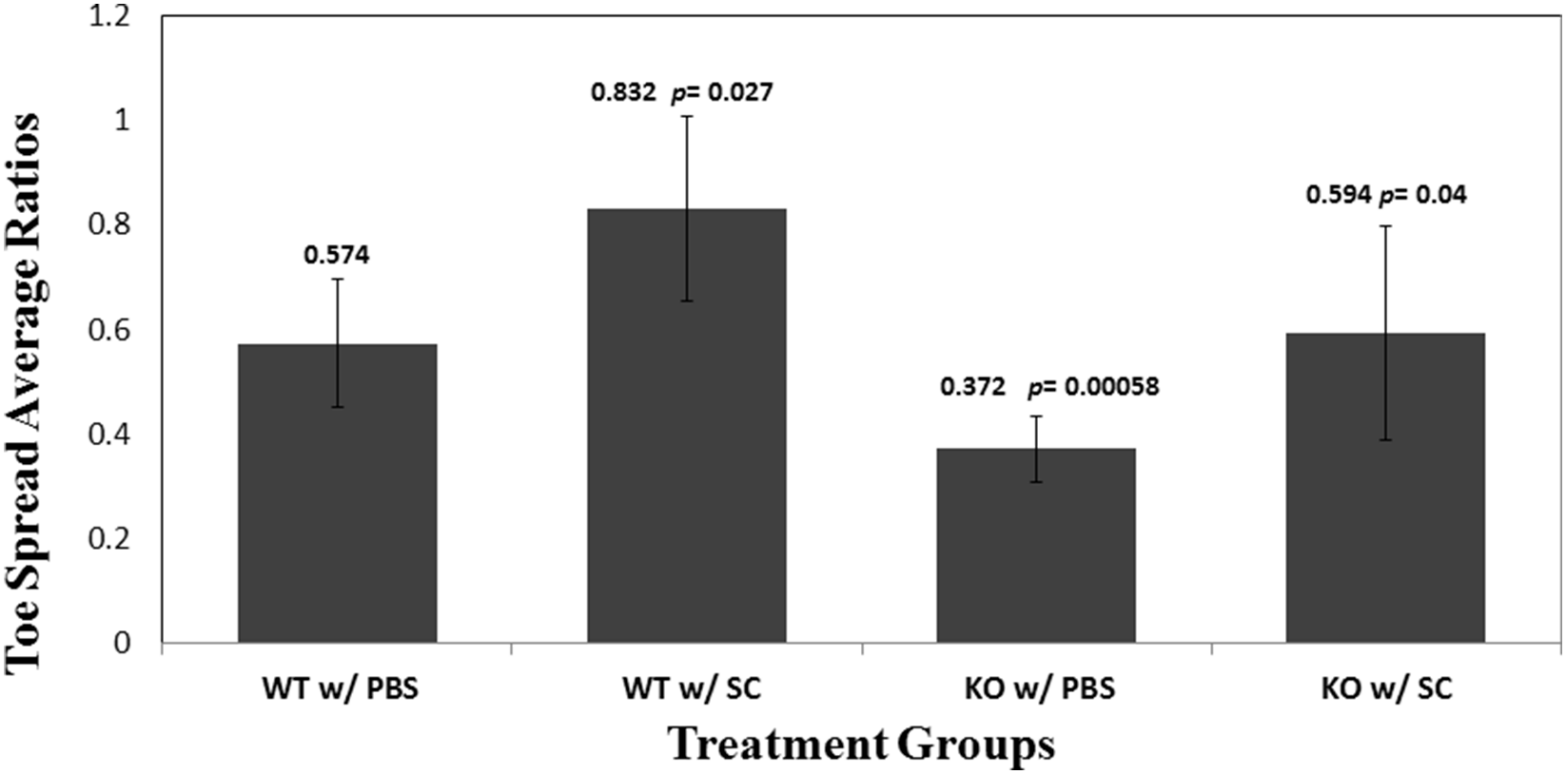
Treatment Effect on Toe Spread Ratio Averages (48–72 hours after treatment). The outcome of stem cell (SC) therapy indicates that IDO may improve recovery. IDO-KO mice treated with SC demonstrated an accelerated recovery compared with IDO-KO treated with PBS (p-value <0.05). However, the WT mice treated with SC showed the greatest recovery of intrinsic paw function when expressed as a ratio comparing it to the non-injured paw (p-value = 0.027). Functional recovery from ischemia-reperfusion (IR) injury in the different treatment groups was measured, using a modified version of walking track analysis. For each subject, toe spread was measured in the IR limb (Ti) and control contralateral limb (Tc). The ratio of the toe spread in the injured limb (Ti) to the control limb (Tc) was then calculated by Ti/Tc. A ratio of 1 indicates 100% recovery or equal width and thus normal intrinsic function. When comparing the WT group treated with stem cells to those treated with PBS, a 45% increase in recovery was seen demonstrating the efficacy of stem cell therapy alone in the presence of an environment where IDO expression is present.

One of the first parameters measured the response each treatment group had from a clinical standpoint. After the IR injury was induced, the mice were observed and videoed to follow their recovery. Forty-eight hours after treatment (72 hours after the initial injury), each mouse, in a blinded manner, was observed looking at their mobility and intrinsic paw function. As described in above, the intrinsic function of each mouse was observed and measured. In the SC treated wild type, the average Ti/Tc was 83.2%. In the wild type with the same IR but no SC, the average Ti/Tc = 57.4%. The improvement by SC in wild type, as measured by toe spread is thus [wild type Ti/Tc with SC]–[wild type Ti/Tc without SC], or 35.8%, and when normalized to the wild type Ti/Tc without SC, by the equation: [Δ Ti/Tc]/[Ti/Tc without SC], or [wild type Ti/Tc with SC – wild type Ti/Tc without SC]/[wild type Ti/Tc without SC] = 35.8/57.4, or 62.4%. In IDO knockout (IDO ko) mice, the same IR injury without SC caused Ti/Tc to decrease to 37.2%. SC was able to increase it in IDO ko group with the same IR injury to 59.4%. [IDO ko Ti/Tc with SC] – [IDO ko Ti/Tc without SC] = 59.4%–37.2% = 22.2%. Using the same equation to calculate the normalized magnitude of improvement, [IDO ko Ti/Tc with SC–IDO ko Ti/Tc without SC]/[IDO ko Ti/Tc without SC] = 22.2/37.2 = 59.7%. The improvement by SC was greater in the wild type (35.8%) compared to the IDO ko group (22.2%) and, though less strong, this still holds when normalized to the controls, 62.4% versus 59.7%. As Figure 1 shows, the WT mice treated with stem cells demonstrated the greatest recovery with 83.2% recovery of intrinsic function of the injured paw to the non-injured paw (*p-value* = 0.0027), compared to the WT group treated with PBS, or a 45% increase in recovery was seen showing the efficacy of stem cell therapy alone in the presence of an environment where IDO expression is present. Additionally, when the WT group treated with SC was compared with its KO counterpart, a 40% increase in recovery (*p-value* = 0.04) is seen.

### Enhancement of Soft Tissues in the Injured Legs

**Figure 2:**
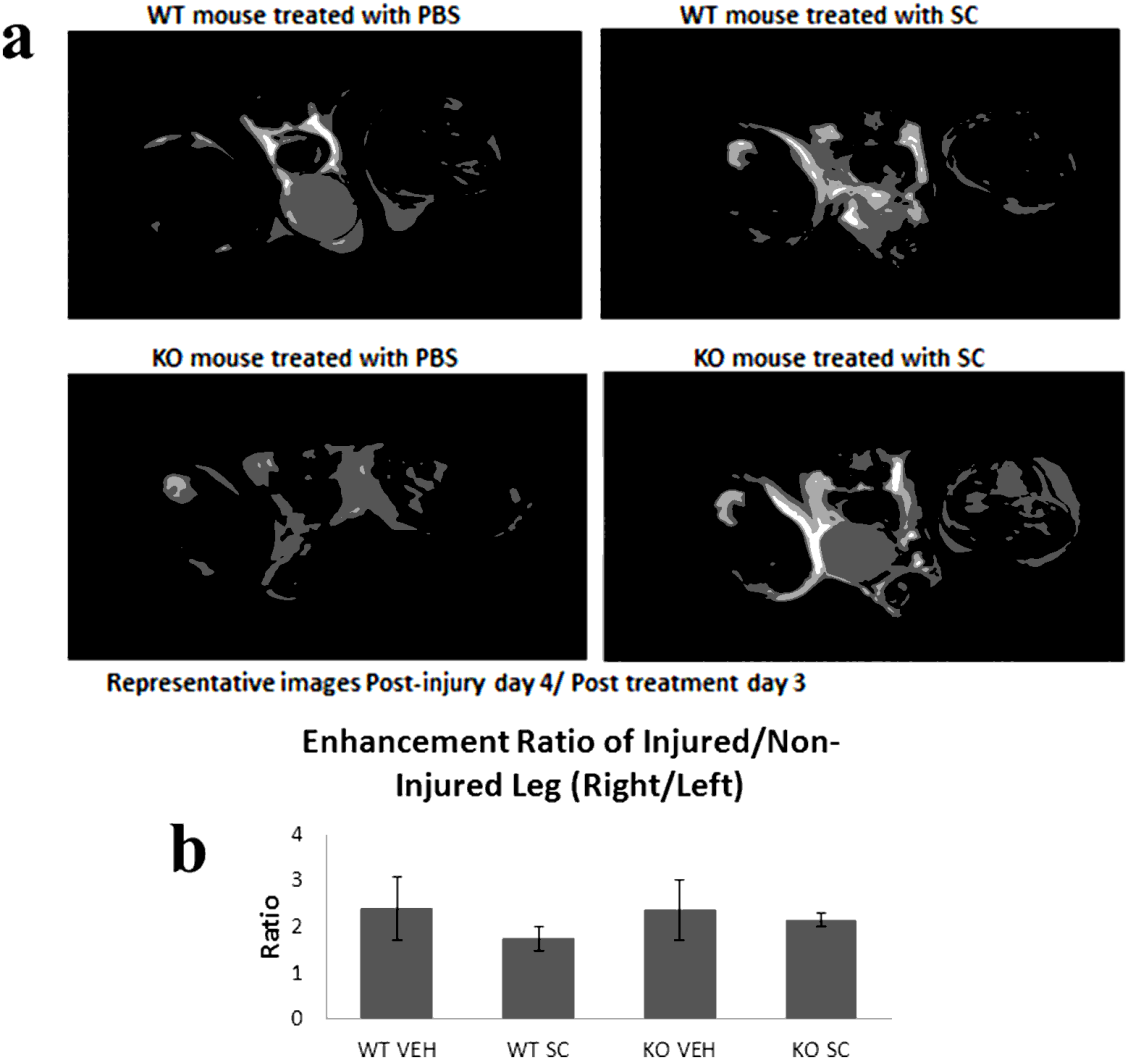
T2 weighted images. **a)** Representative T2 weighted imaging cuts where the enhancement of the soft tissue can be observed within the injured legs and compared to the non-injured legs and, **b)** The average ratio of enhancement measured between the injured leg over the non-injured leg are shown in this representative graph for each of the 4 groups.

As Figure 2a shows, enhancement of the soft tissue can be observed within the injured legs compared to the non-injured legs. Image J was used to calculate the enhancement seen in the soft tissues within the injured leg. As shown in Figure 2b, WT mice treated with stem cells showed the lowest ratio of soft tissue enhancement, comparing the injured to non-injured leg, indicating the stem cell therapy in the WT mice had the greatest ability to recover.

### Stem Cells Enhance IDO Expression in Tissue Microenvironment

**Figure 3:**
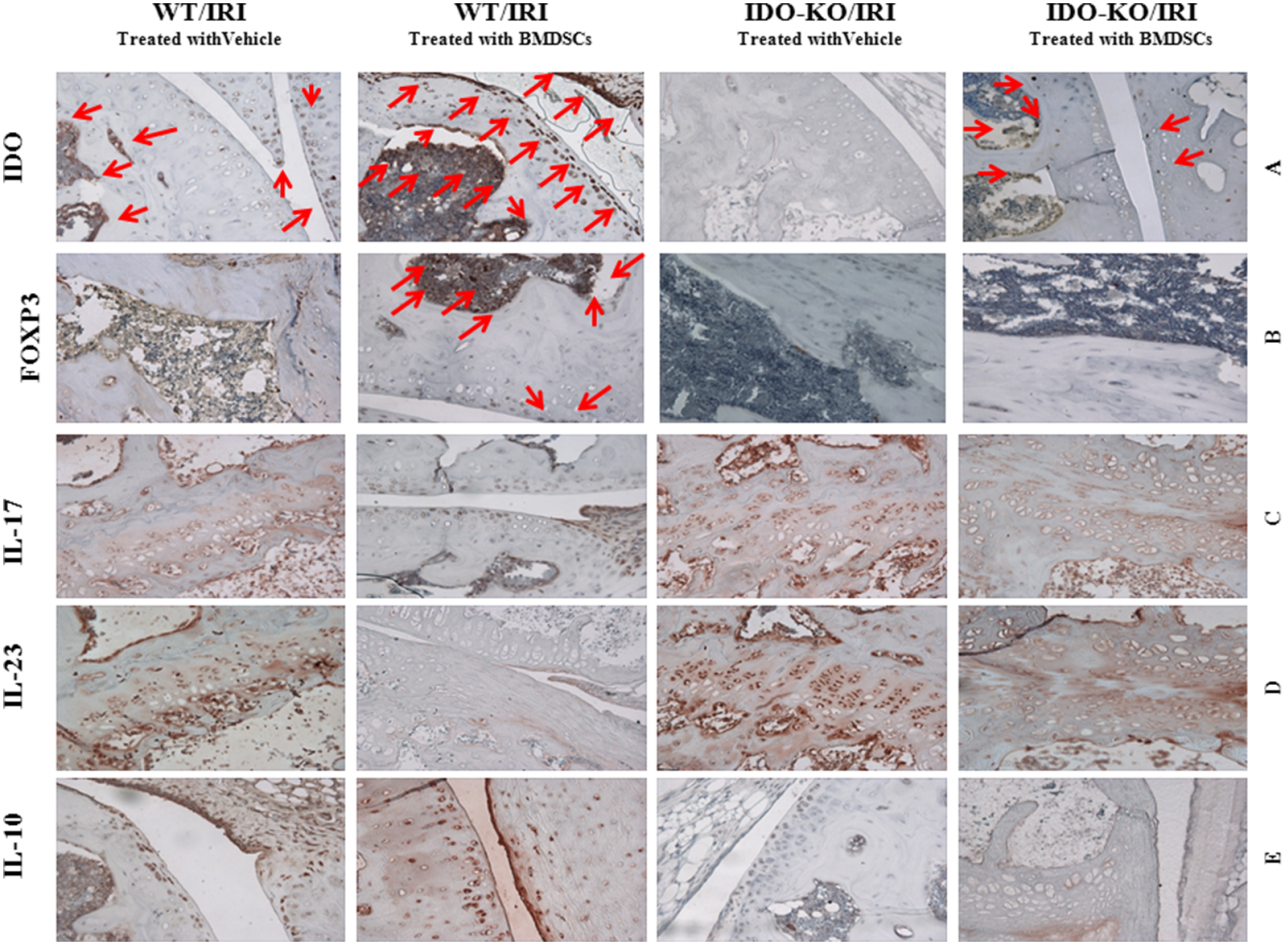
BMDScs can enhance IDO and regulatory T cells while reducing inflammatory cytokines in the hind limb IR injury. Immunohistochemical analysis of paraffin embedded tissues from murine model with IRI of hind limb showed that treating the animals with BMDSCs in an IDO sufficient microenvironment first: increased IDO and FOXP3 expression (panels A and B, red arrows), while decreased the inflammatory cytokines, IL-17 and IL-23 (panels C and D). Anti inflammatory cytokine, IL-10, was increased as demonstrated in panel E. All together, these analysis suggest a potential therapeutic role for BMDSCs, re-enforced by possible IDO dependent mechanisms. All pictures are 400X magnification.

As demonstrated in Figure 3 (panel a), immunohistochemical assessment indicated the fact that IDO expression was increased in WT mice treated with SCs after injury compared to other groups. Indeed, as shown in Figure 3, enhancement of IDO expression is mediated by SCs as evidenced by both higher expression of IDO in treated WT compared to non-treated WT, as well as by minimum expression of IDO in IDO KO animals treated with SCs compared to no expression of IDO in non-treated IDO KO animals (as SCs were the only source of IDO in these KO recipient animals). These results suggest that SCs are inserting their regulatory effects into the tissue microenvironment through a variety of mechanisms of which, enhancement of IDO may potentially be a crucial determinant of recovery in a hind limb IR injury.

### Stem Cells Attenuate Inflammation with their Most Profound Effects in the Presence of IDO

#### Immunohistochemical analysis

Consistent with our clinical observation and imaging results, histological and immunohistocheical analysis demonstrated the significant reduction in the expression level of IL-17, IL-23 in WT mice treated with SCs compared to the other experimental groups (Figure 3, panels C and D). Meanwhile, the expression level of FOXP3 (Treg marker) and IL-10 were increased in WT mice treated with SCs compared to the other experimental groups (Figure 3, panels B and E). These results suggest that IDO may be an effective factor in the efficiency and beneficial impact of SCs in a hind limb IRI setting, which may imply a therapeutic application based on SCs and enhancement of IDO.

#### Analytical flow cytometry

**Figure 4:**
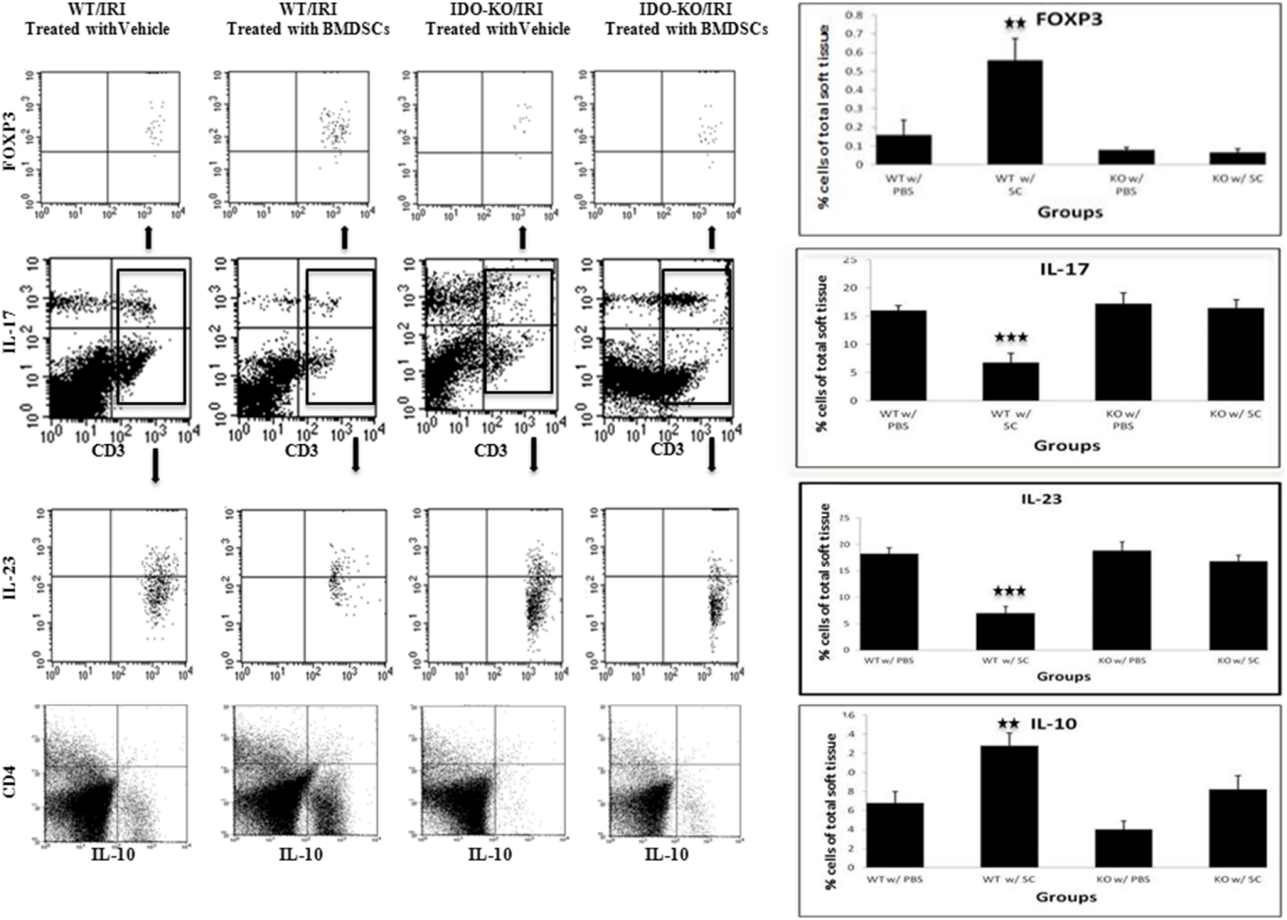
Regulatory effects of SCs may be partially associated with IDO presence in tissue microenvironment. Dot Plots are representatives of flow cytometric analysis of live single cell preparations from murine hind limb tissues with IRI showed that in the presence of IDO, injection of BMDSCs decreased CD3+ cells (T cells), IL-17 and IL-23 expression (IL-23 is based on gating of IL-17+ cells). Further, applying BMDCs could increase both CD3+ FOXP3+ cells (Tregs, gated on CD3+ cells) and IL-10 expression in an IDO sufficient microenvironment. Bargraphs on the right side of each group are reflecting the accumulative analysis of five animal per experimental group. Statistical analysis was performed using Kruskal-Wallis test with Dunn’s multiple-comparison post- hoc test. * = p<0.05, ** = p<0.01, *** = p<0.001.

As demonstrated in Figure 4, in accordance with our histological data and consistent with our clinical observation, flow cytometry analysis showed WT mice treated with stem cells attenuate the injury-induced inflammatory response (*p-value*<0.04). Figure 4 shows that the expression of cytokines IL-17, well-established marker for inflammation derived from T-helper-17 (Th-17) cells, and IL-23, a powerful pro-inflammatory cytokine, was lower in the tissue from the WT mice treated with stem cells than any other group, while in IDO KO group, both IL-17 and IL-23 expression were the highest. More importantly, the regulatory T cells (FOXP3+T regs), anti-inflammatory index, were detected 8.75 times higher in the WT mice treated with SC compared to the KO group treated with SC. Moreover, IL-10, an anti-inflammatory cytokine, was 56% higher in the WT group treated with SC when compared to its KO counterpart at 12.8% (compared to 8.2%) (Figure 4c). These findings reaffirm the important anti-inflammatory effects of stem cell therapy and the critical role IDO plays in the efficacy of stem cell therapy.

### Stem Cells Reduces Cell Death with their Most Profound Effects in the Presence of IDO

**Figure 5:**
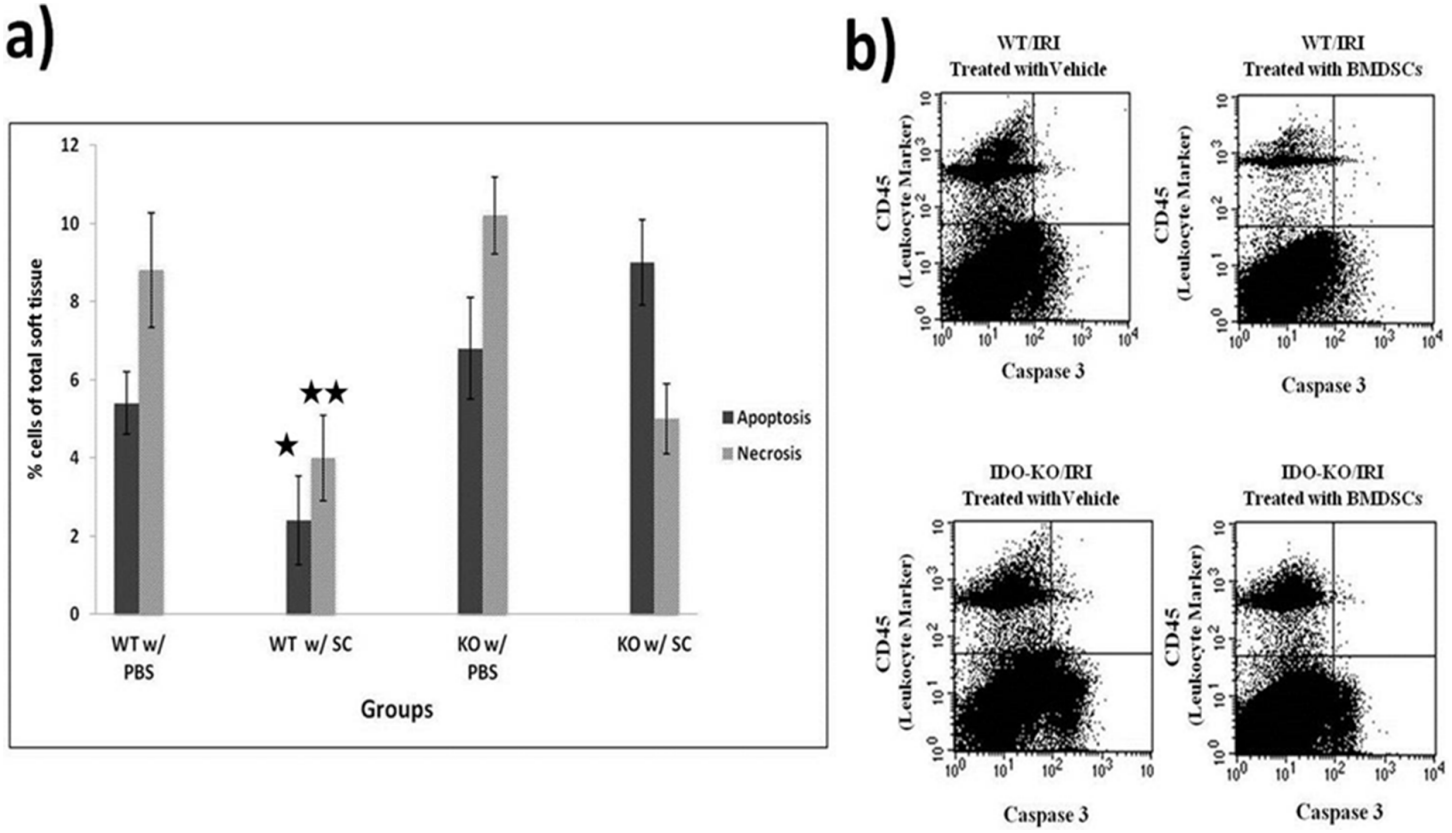
SCs treatment reduces cell death in an IDO dependent manner. Flow cytometry analysis showed (5a) that stem Cells reduce cell death (apoptosis/necrosis) with their most profound effects in the presence of IDO. Further, flow cytometric analysis demonstrated that a majority of reduction in cell death occurred among parenchymal cells (CD45 negative) and not infiltrated leukocytes (CD45+), indicating the beneficial impact of SCs in reducing cell death and necrotic responses, leading to less inflammation, more protection and faster recovery. Statistical analysis was performed using Kruskal-Wallis test with Dunn’s multiple-comparison post- hoc test. * = p<0.05, ** = p<0.01, *** = p<0.001.

As shown in Figure 5a, while an IR insult resulted in a marked increase in cell death (both apoptosis and necrosis) in the ischemic-reperfused limbs, treatment with SCs resulted in a marked decrease in cell death in the ischemic-reperfused limbs with a higher rate of reduction in the presence of IDO compared to IDO KO mice. Using flow cytometry analysis, markers for necrosis (7-aminoactinomycin D, 7-AAD) and apoptosis (Caspase 3) were measured and consistent with all results shown above. The lowest levels of both markers were seen in the WT group treated with SC by a considerable margin when compared to the corresponding groups and highest in the two KO groups. When analyzing data for Caspase 3, the WT group treated with stem cells showed an average of 2.4% apoptotic expression compared to 5.4% in the vehicle treated WT group and 9% in the KO group treated with stem cells. The studies of necrosis showed similar trends with an average of 4% necrosis in the WT treated with stem cells group, while the vehicle treated WT mice showed an average of 8.8% cell necrosis and the vehicle treated KO group up to 10.2% average cell necrosis. Importantly, as is showed in Fig. 5b, CD45 (a marker to differentiate the hematopoietic cells from non-hematopoietic ones), was used to show that the effect of SCs cell on reducing apoptosis/necrosis was mainly on non-hematopoietic cells, leading to restoring tissue viability, healing and protection. These findings suggest that the adverse effects of ischemia reperfusion can be reduced by insertion of SCs into an IDO sufficient micro-environment, resulting in less cell death and faster recovery, supporting our hypothesis that IDO may play a critical role in enhancing the beneficial effects of stem cell therapy.

## Discussion

We demonstrated that intramascular injections of BMDSC decrease the recovery time after IR injury in hind limb ischemia reperfusion injury. Most importantly, our results suggest that IDO modulatory feature may play a potential role in beneficial effects of BMDSCs in an ischemia reperfusion setting of hind limb injury, manifested by marked anti-inflammatory effect of these cells and reduction of apoptosis/necrosis inside the tissue microenvironment.

Ischemia reperfusion injury results from the culmination of events starting with the initial ischemic insult which leads to metabolic, cellular, immunologic and environmental changes and ultimately inflammation and cell death [2], [24], [25]. The immunologic response, including the release of inflammatory mediators and recruitment of cells, can be a key to modulating the response, and ultimately the degree of damage and cell death seen as the result of a given insult. The immunologic significance of IDO has been shown over the years from its involvement in host defense against pathogens, tumor immune evading properties and embryo survival from the mother’s immune responses to name a few [17]–[20], [26]–[34]. Our goal was to better understand IDO’s role in the immunologic response seen with IR injury as it relates to stem cell therapy. This knowledge can be used to favorably decrease the injury which is extremely valuable given the myriad of clinical conditions by which such therapeutic potentials can be used. Stem cell therapy has been described to lessen the inflammatory response and injury that results from IR through several mechanisms including paracrine and autocrine signaling, neutralization of ROS, promotion of angiogenesis, and modulation of the innate and adaptive host responses as described previously [4], [5], [9], [11], [12], [35], [36]. Our results bring to the forefront the novel idea that IDO, a known modulator of the immune response pathways, plays a critical role in the anti-inflammatory effects seen with stem cell therapy in the setting of IR injury. One approach that is readily translatable into clinical use is the up-regulation of IDO by small stable compounds such as CpG oligodeoxynucleotides (CpG-ODNs) alone or in conjunction with BMDSCs. CpG-ODN has been shown since 2005 to increase IDO-dependent T-regulatory cell function [28], [38], [39]. Any ischemic event, such as organ transplantation, revascularization after myocardial infarction, stroke, limb re-vascularization, and micro-vascular tissue transfer, CpG-ODNs can be given IV after blood flow is restored to reduce the effects of IR injury induced inflammation.

Stem cell use for clinical application has been researched extensively, including for IR injury. However, the ability to control stem cell response with consistent results has been unsuccessful, in part, because of an incomplete understanding of how they confer their effects. In the setting of IR injury using a murine model, our experiments showed injury to the hind limb improved more rapidly and returned to more normal intrinsic function in both the WT and KO groups when compared to their vehicle treated counterpart. Using the non-injured leg as a relative control and indication of both baseline function, and tissue properties, clinically observed intrinsic function and tissue inflammation could be compared across all groups. We consistently found that stem cell therapy, especially in the presence of IDO, greatly improved both the intrinsic function observed and attenuated the inflammatory response, and therefore lessened the soft tissue edema seen in the T2 weighted studies. These observations are congruent with previous studies citing the anti-inflammatory properties of BMDSCs.

In order to further investigate the anti-inflammatory effects of BMDSC therapy appreciated in our MRI studies, we also performed a variety of IHC and flow cytometry studies. The inflammatory response as indicated by the expression of IL-17 and the anti-inflammatory markers IL-10 and FoxP3+ confirmed the anti-inflammatory properties of stem cell therapy, especially in the WT group. The attenuated inflammatory response as indicated by decreased expression of IL-17 and increased IL-10 and FoxP3+ also seems to have a direct effect on the end result of cell death as indicated by the percent of cells undergoing apoptosis and necrosis. Our data showed that most of the IL-10 expressing cells were non-CD4 cells, suggesting that other type of cells rather than CD4+ cells, including (not limited to) bone marrow derived stem cells, CD8+ lymphocytes, as well as dendritic cells may be the potential source of IL-10. Several reports previously have demonstrated the production of IL-10 by SCs, monocytes and non-CD4 lymphocytes [41]–[43]. However, these findings provide additional evidence that SCs may alter cytokine production profile from a pro-inflammatory (e.g., IL-17) to an anti-inflammatory (e.g., IL-10) status in a paracrine/autocrine fashion.

The observations of improved clinical recovery decreased tissue edema, attenuated markers of inflammation and decreased overall cell death seen with BMDSCs and especially in the WT group which bring us to our last point about the critical role IDO plays in this process.

In studies from both our team and others, IDO has shown to be a vital enzyme used by cells to modulate the host immune response depending on the environmental ques. In instances of pathogenic insults, IDO has been shown to help up-regulate the immune response in a host protective manner. During pregnancy, IDO expression seen in trophoblasts surrounding the embryo have been shown to be crucial to maintaining the embryo and protecting it from T cell mediated rejection [37]. As cited earlier, many other experiments have shown the duality of IDO which is dependent on the timing of expression. The cells which express it and the micro environmental conditions at the time of expression help determine the effects its expression has on immune response. In the experiments performed during this study, we have repeatedly shown that the effects of SCT therapy have an impressive anti-inflammatory effect with down-regulation of inflammatory mediators including IL-17, IL-23 and increased expression of anti-inflammatory index IL-10 and FoxP3+. Additionally, the percent of cells (non-hematopoitic cells) undergoing apoptosis and necrosis at 48–72 hours after BMDSC treatment in the WT group were less than half of any other group and correlate well with the clinical findings of improved recovery, mobility and intrinsic function. Additionally, these findings were supported with MRI T2 weighted studies showing less edema and inflammation within the soft tissues of the stem cell treatment mice compared to the non-treated mice and a significant difference (p<0.05, n = 4, using 2 tailed t-test) between the IDO WT and KO mice treated. The constellation of these findings suggests the protective effects of SCT are critically dependent on the expression of IDO to induce its anti-inflammatory effects. Without IDO present in the environment, inflammation induced by the IR injury is more pronounced and so is necrosis and apoptosis of the tissues, which leads to a longer recovery time seen clinically.

The future of IDO for clinically relevant therapeutic interventions and the responsible mechanisms need to be investigated in more detailed and comprehensive studies. Antibiotics cannot be developed before we understand the properties of the pathogen and how it attacks the host. Similarly, the most important step toward being able to tap into IDO’s therapeutic potential is by understanding the mechanisms and principles, which it modulates and interacts with the immune system during various clinical scenarios. Teasing out the mechanisms of cross talk between different intra and intercellular pathways during inflammatory conditions like IR and relating them in response to new ideas and findings such as IDO will allow us to more appropriately and effectively apply these concepts towards new therapies. These studies were done to help define the importance of IDO’s presence during stem cell therapy for IR injury, but its translation is not limited to SCT alone, but all incidents were IDO and inflammation are involved. IDO has been linked to a tumors ability to remain immune tolerant and so modulation of IDO expression could potentially help therapeutic measures like chemotherapy and radiation be more effective in our ability to fight oncological disease. Ultimately, allograft transplantation fails in the long term due to a slow, T-cell mediated rejection of the organ. It has been discussed that IDO inducers and enhancement within the tissue can help protect the organ from such rejection much in the same way trophoblasts protect the embryo from the mother’s immune system [28], [38], [39]. With continued efforts to more profoundly understand the functions of IDO and how it influences states of inflammation and immune response, the potential clinical implications are vast and deserve further intense investigation.

In conclusion, the beneficial anti-inflammatory effects of SCs may be partially actuated through an IDO dependent mechanism, which not only shortens the recovery time, but also confers significant protection against cell death in the ischemic re-perfused limb. These effects were associated with increased recruitment and mobilization of Tregs as well as regulatory cytokines such as IL-10. Collectively, the results suggest that the immune-modulatory role of IDO in SCs may offer a novel approach to protecting the hind limb against acute injury secondary to an IR insult. However, the novel demonstration of the prominent impact of IDO on mobilization and function of stem cells is not only of clinical relevance and significance for the hind limb ischemia reperfusion injury, but for other immune and inflammatory disorders, including organ transplant and cancer.
